# Molecular Characterization of Culturable Aerobic Bacteria in the Midgut of Field-Caught *Culex tritaeniorhynchus*, *Culex gelidus*, and *Mansonia annulifera* Mosquitoes in the Gampaha District of Sri Lanka

**DOI:** 10.1155/2020/8732473

**Published:** 2020-10-01

**Authors:** Nayana Gunathilaka, Koshila Ranasinghe, Deepika Amarasinghe, Wasana Rodrigo, Harendra Mallawarachchi, Nilmini Chandrasena

**Affiliations:** ^1^Department of Parasitology, Faculty of Medicine, University of Kelaniya, Ragama, Sri Lanka; ^2^Department of Zoology and Environmental Management, Faculty of Science, University of Kelaniya, Colombo, Sri Lanka; ^3^Biotechnology Unit, Industrial Technology Institute, Colombo 07, Colombo, Sri Lanka; ^4^Department of Parasitology and Medical Entomology, Medical Research Institute, Colombo 08, Colombo, Sri Lanka

## Abstract

**Background:**

Larval and adult mosquito stages harbor different extracellular microbes exhibiting various functions in their digestive tract including host-parasite interactions. Midgut symbiotic bacteria can be genetically exploited to express molecules within the vectors, altering vector competency and potential for disease transmission. Therefore, identification of mosquito gut inhabiting microbiota is of ample importance before developing novel vector control strategies that involve modification of vectors.

**Method:**

Adult mosquitoes of *Culex tritaeniorhynchus*, *Culex gelidus*, and *Mansonia annulifera* were collected from selected Medical Officer of Health (MOH) areas in the Gampaha district of Sri Lanka. Midgut lysates of the field-caught non-blood-fed female mosquitoes were cultured in Plate Count Agar medium, and Prokaryotic 16S ribosomal RNA partial genes of the isolated bacteria colonies were amplified followed by DNA sequencing. Diversity indices were used to assess the diversity and richness of the bacterial isolates in three mosquito species. The distribution pattern of bacterial isolates between different mosquito species was assessed by Distance-Based Redundancy Analysis (dbRDA).

**Results:**

A total of 20 bacterial species (*Staphylococcus pasteuri*, *Bacillus megaterium*, *Staphylococcus cohnii*, *Pantoea dispersa*, *Staphylococcus chromogenes*, *Bacillus aquimaris*, *Staphylococcus arlettae*, *Staphylococcus sciuri*, *Staphylococcus warneri*, *Moraxella osloensis*, *Enterobacter* sp., *Klebsiella michiganensis*, *Staphylococcus hominis*, *Staphylococcus saprophyticus*, *Streptomyces* sp., *Bacillus niacin*, *Cedecea neteri*, *Micrococcus luteus*, *Lysinibacillus sphaericus*, and *Bacillus licheniformis*) were identified. All of these species belonged to three phyla, *Proteobacteria*, *Firmicutes*, and *Actinobacteria*, out of which phylum *Firmicutes* (71.1%) was the most prominent. The least number of species was recorded from *Actinobacteria*. The relative distribution of midgut microbes in different mosquito species differed significantly among mosquito species (Chi-square, *χ*^2^ = 486.091; *df* = 36; *P* ≤ 0.001). Midgut microbiota of *Cx. tritaeniorhynchus* and *Cx. gelidus* indicated a similarity of 21.51%, while *Ma. annulifera* shared a similarity of 6.92% with the cluster of above two species. The gut microbiota of *Cx. tritaeniorhynchus* was also significantly more diverse and more evenly distributed compared to *Ma. annulifera.* Simpson's diversity, Margalef's diversity, and Menhinick's diversity indices were higher in *Cx. gelidus*. Of the recorded species, *P. dispersa* and strains of nonpathogenic species in *Bacillaceae* family (*B. megaterium*, *B. niacini*, *B. licheniformis*, and *L. sphaericus*) can be recommended as potential candidates for paratransgenesis.

**Conclusion:**

The relative distribution of midgut microbes in different mosquito species differed significantly among the three studied adult mosquito species. The present data strongly encourage further investigations to explore the potential usage of these microbes through paratransgenic approach for novel eco-friendly vector control strategies.

## 1. Background

Mosquitoes transmit a wide range of pathogens that cause diseases in humans and other animals. All mosquito species are aquatic during the immature stages [1]. Studies conducted in the early 1900s noted that larval and adult stages of mosquitoes harbor extracellular microbes in their digestive tract [2, 3]. During the last 10 years, their roles for mosquito biology have been broadly studied. Some findings emphasize that they play important roles in immunity, food digestion, fertility, and fecundity, which ultimately affect larval growth, adult fitness, vector populations, and disease prevalence [4].

Besides, these studies evidenced positive and negative effects of these gut microbial communities on vector competency through host-parasite interaction [5, 6], thereby significantly influencing disease transmission potential [7, 8]. Moreover, mosquito-midgut bacteria are used as vehicles to express molecules in the gut and suppress parasite colonization [9, 10]. This technique is known as paratransgenesis, which emerged as a novel vector controlling strategy. Here, the symbiotic bacteria associated with vectors may genetically modify for the expression of an effector molecule and reintroduced through food to the mosquito midgut.

The identification of suitable midgut bacteria is the fundamental requirement in the paratransgenic techniques. These identified organisms should be able to colonize in the midgut of an interested disease vector and should be transferred to the next generation through transstadial passage [11]. The number of recent studies has used culture-dependent and culture-independent approaches to characterize the microbial communities in the midgut of different mosquito species including *Aedes*, *Culex*, *Anopheles*, *Coquillettidia*, and *Mansonia* [12–17]. However, little is known about the midgut microflora of *Culex* mosquitoes, and very few studies have been conducted on midgut microbiota [11, 18, 19]. In addition, no studies have been carried out to screen the midgut bacteria in *Mansonia annulifera* mosquitoes.

The mosquito *Culex tritaeniorhynchus* is the major vector of Japanese encephalitis (JE) virus throughout Asia and the pacific islands including Sri Lanka [20–23]. The JE has occurred in Sri Lanka for decades, seriously threatening human life since 1980 [21]. Gampaha was one of the districts that had reported a relatively large number of JE cases in the past [24]. The success of the immunization against JE in Sri Lanka is reflected in the fact that since 1988, the incidence of JE has decreased drastically with the increased coverage of immunization. However, the disease was spreading to new areas with previously low levels of enzootic transmission and report occasional outbreaks in some districts in Sri Lanka [25].

On the other hand, *Ma. annulifera* is a vector of brugian filariasis and dirofilariasis in Sri Lanka [26]. Until recently, brugian filariasis was considered an infection of the past as cases were not reported in Sri Lanka since the late 1960s. However, surveillance activities carried out to evaluate the Programme for Elimination of Lymphatic Filariasis (PELF) in Sri Lanka revealed the sporadic occurrence of brugian filariasis [27, 28] of probable zoonotic origin [29] in endemic areas. In 2016, Sri Lanka was certified to have eliminated lymphatic filariasis as a public health problem by the World Health Organization [30]. However, the occurrence of zoonotic brugian filariasis raises an issue as mass treatment of the human population with antifilarial drugs would not eliminate the reservoir of infection nor the vector population. Human dirofilariasis is another zoonotic filarial infection, commonly encountered in the country [31–33].

The abundance of disease-transmitting vectors due to limited vector control activities and higher receptivity in these areas have been identified as major challenges to maintain the disease-free status in the country, for JE and filariasis. Therefore, alternative vector control methods are needed as additional tools to be incorporated into the integrated vector management [32]. Paratransgenesis could be a better control tool for vector management. Hence, the present study was conducted to determine the microflora in the midgut of wild-caught *Cx. tritaeniorhynchus*, *Cx. gelidus*, and *Ma. annulifera* which has been limitedly assessed world-wide.

## 2. Methods

### 2.1. Selection of Study Area and Sampling Sites

**Figure 1:**
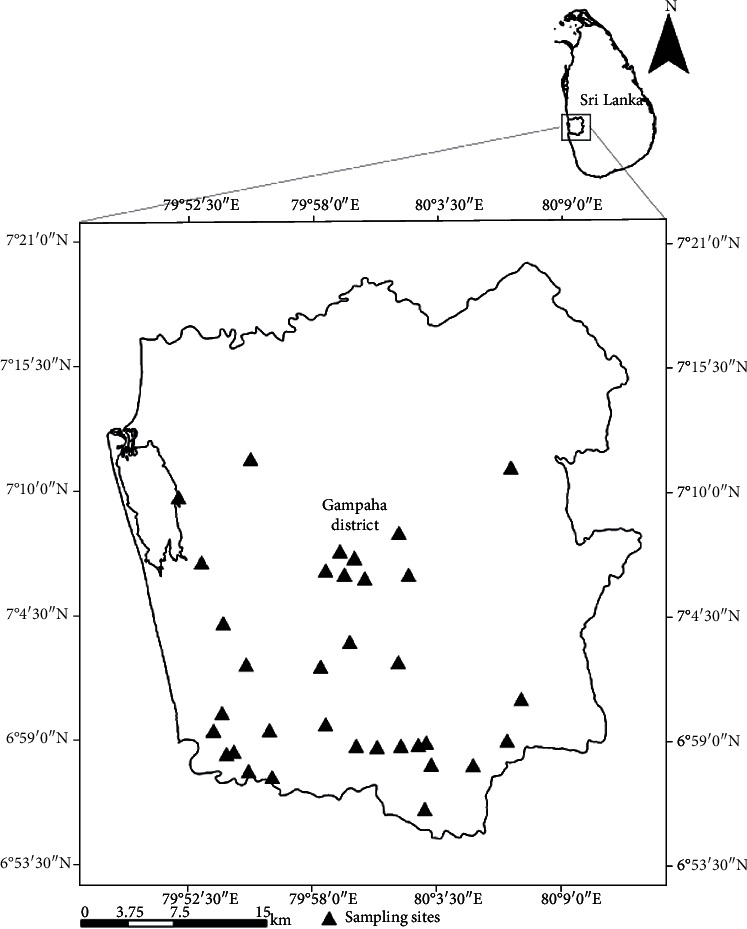
The GIS map showing the geographical distribution of mosquito sampling sites in Gampaha District, Sri Lanka.

The study was conducted in the district of Gampaha which is endemic for lymphatic filariasis, dirofilariasis, and JE diseases in Sri Lanka. This district is the second most populous district in Sri Lanka with urban, semiurban, and rural populations in a land area of 1387 km^2^ [34]. Sampling sites were selected from Kelaniya (06° 58.426′ N, 79° 54.939′ E) and Gampaha (07° 06.448′ N, 79° 53.049′ E) Medical Officer of Health (MOH) areas to carryout mosquito collections (Figure 1). The selection of other sample sites was carried out in consultation with the Regional Entomologist, Gampaha District, based on the vector abundance and potential risk for filariasis with reference to the past prevalence rates of microfilaraemia/antigenaemia.

### 2.2. Field Collection of Adult Mosquitoes

Entomological surveys were conducted at each selected location from January to October 2019 using cattle-baited net trap (CBNT) collections according to the standard guidelines described by the World Health Organization [35]. The cattle-baited net traps were set up at each selected site in the evening (17:00-18:00 h). The trap was made of a white cotton drill (3 m × 3 m × 1.5 m) with net windows (2 m × 1 m) on sides erected using a strong center pole of two-meter height and four side sticks each of the same height (1.5 m). A distance of 15-25 cm gap was allowed between the lower edge of the net and the ground, enabling the mosquitoes to enter the trap. The cattle was introduced into the trap just before the sunset and tethered to the pole fixed to the mid of the trap. The mosquitoes were collected from 21:00-23:00 in the late-night using battery-operated aspirators. Collected adult mosquitoes were transferred safely to the presterilized adult rearing cages. All collected adult mosquito samples were transported and housed at the insectary facility at the Department of Parasitology, Faculty of Medicine, University of Kelaniya, Ragama, Sri Lanka, until taken for further experiments.

### 2.3. Processing and Identification of Field-Caught Adults

Unfed (non-blood-fed) live mosquitoes captured from the field that housed at the insectary were sorted within 6-8 hours after collection. The mosquitoes were sacrificed using a clod shock. Mosquito species, namely, *Culex tritaeniorhynchus*, *Culex gelidus*, and *Mansonia annulifera*, were segregated based on key morphological characteristics [36–38]. Mosquito adults were then surface sterilized individually for 30 seconds by tapping in a microcentrifuge tube containing 250 *μ*L of 70% ethanol followed by rinsing twice with 250 *μ*L of phosphate-buffer saline (PBS). The final discard of washing was used for bacterial analysis to make sure that there was no bacterial contamination from the mosquito surface. For isolation of the midgut bacterial population, the midguts of non-blood-fed adult female mosquitoes were dissected under sterile conditions. Blood or sugar feeding was not performed during the rearing process before recruitment for midgut analysis, to avoid the potential alterations in the midgut microflora.

Individually dissected midguts of five adults were pooled and transferred to a 1.5 mL microcentrifuge tube containing 250 *μ*L of PBS and homogenized with a sterile micropestle. The homogenized lysate was serially diluted in PBS (900 *μ*L) to prepare a dilution series from 10^0^-10^−7^. From each mosquito species, a minimum of 250 adults was processed separately to screen for gut flora.

### 2.4. Culturing and Isolation of Bacteria

A volume of 100 *μ*L from each dilution was plated on sterile Plate Count Agar (PCA) media and incubated at 35°C for 24-48 hours. To assess microbial growth, the total number of Colony-Forming Units (CFUs) was determined. Bacterial colonies obtained on plates were differentiated morphologically on colony shape, size, color, margin, opacity, elevation, etc. Morphologically distinct colonies were selected from primary plates and subcultured on nutrient agar plates until a pure colony was obtained. All bacterial isolates were identified up to the genus level by Gram-staining and biochemical testing performed according to Cowan and Steel's manual [39]. Finally, the bacterial isolates differentiated up to the genus level by Gram-staining and biochemical testing were selected further for molecular characterization.

### 2.5. Genomic DNA Extractions and Sequencing

Genomic DNA was isolated from each pure culture using QIAamp DNA mini kit (QIAGEN GmbH, Hilden, Germany) according to the manufacturer's instructions. The PCR amplifications were performed using universal primers 27F (5′ AGAGTTTGATCCTGGCTCAG 3′) and 1492R (5′ TACGGCTACCTTGTTACGACTT 3′) [40] targeting 16S rRNA gene sequences. Polymerase Chain Reaction (PCR) was performed with a reaction mixture containing 1x PCR buffer (Invitrogen), 0.5 *μ*M of each primer, 2.5 mM MgCl_2_, 200 ng of purified DNA, 0.2 mM dNTPs, and 0.3 units of *Taq* polymerase (Invitrogen). The total volume was adjusted to 25 *μ*L. The PCA media and ddH_2_O were used as negative controls. Samples were amplified according to the following cycle: initial denaturation at 94°C for 10 min, followed by 35 cycles of denaturation at 94°C for 30 s, annealing at 55°C for 30 s, and extension at 72°C for 1 min. The final extension was at 72°C for 8 min.

The amplified product was visualized on a 1% agarose gel containing ethidium bromide using a UV transilluminator. The PCR amplicons were then purified using the QIAquick PCR Purification Kit (Qiagen). The purified products were sent to Macrogen, South Korea (Macrogen Inc., 1001, 254 Beotkkot-ro, Geumcheon-gu, Seoul, Republic of Korea) for 16S ribosomal RNA partial gene sequencing by the Sanger method.

### 2.6. Phylogenetic Analysis of Midgut Bacteria Isolated from Mosquito species

Homologous sequences were searched in the GenBank database using BLAST [41]. The isolates were identified when their 16S rRNA gene sequences shared 97% homology with completed 16S rRNA gene sequences found in the GenBank database. The evolutionary history was inferred using the neighbor-joining method, and the evolutionary analyses were conducted in MEGA X. The evolutionary distances which were computed using the Tajima-Nei method were used to infer the phylogenetic tree.

A phylogenetic tree was also constructed for assessing the phylogenetic affiliations of bacterial isolates detected in the midguts of three selected mosquito species and with the usage of some reference sequences available in the literature [19]. Phylogenetic branch length distance (BLD) was estimated based on the maximum likelihood (ML) tree which is estimated on the multiple sequence alignment (MSA). The dissimilarity between a pair of sequences was defined as the total branch length distance on the ML tree between a pair of sequences.

### 2.7. Data Analysis

The percentage relative abundance of each mosquito species was calculated. The Excel spreadsheet tool [42] was used to calculate confidence intervals for the comparison of the presence of each bacterial species in mosquito species. The Chi-square test of independence was used to evaluate the significance in the distribution of different bacteria species in different mosquito species. Diversity indices such as the Simpson Index [43], Shannon Index [44], Margalef diversity index [45], Menhinick's diversity index [46], and evenness of bacterial communities [47] in each tested mosquito were calculated. All the interpretations were done considering 95% significant intervals.

## 3. Results

### 3.1. Bacterial Isolates from the Midgut of Different Mosquito Species

A total of 20 bacterial strains were identified from the midgut of three selected vector mosquitoes. The majority of the midgut isolates (71%; 13/20) were categorized under the phylum *Firmicutes*. The other seven species were from the *Proteobacteria* (18.7%; *n* = 5) and *Actinobacteria* (10.19%; *n* = 2) phyla. The list of bacteria species identified from the midgut lumens of each mosquito species is illustrated in Table 1.

### 3.2. *Cx. tritaeniorhynchus*

**Figure 2:**
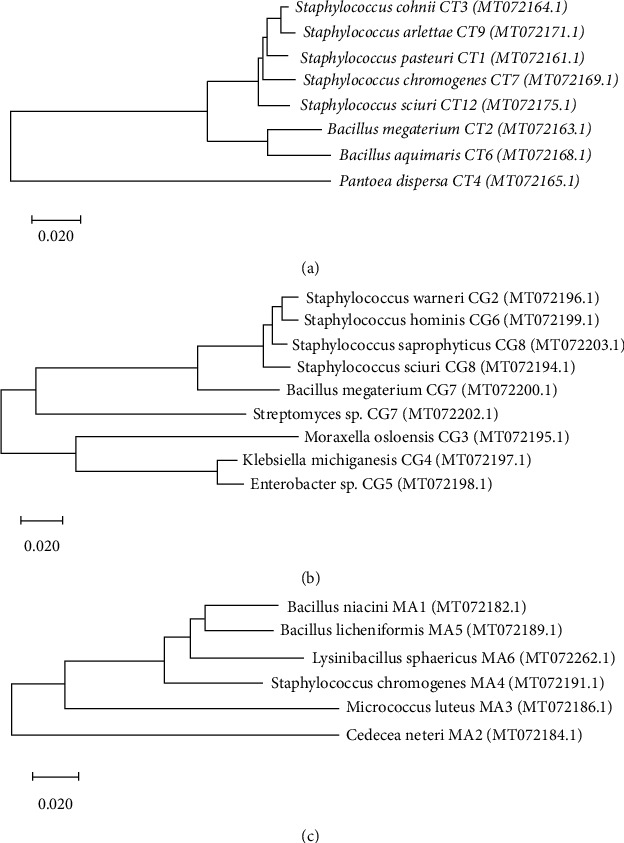
Dendrogram showing phylogenetic affiliation of bacterial isolates in the midgut of (a) *Cx. tritaeniorhynchus*, (b) *Cx. gelidus*, and (c) *Ma. annulifera.*

A total of eight different bacterial species, namely, *Staphylococcus pasteuri*, *Bacillus megaterium*, *Staphylococcus cohnii*, *Pantoea dispersa*, *Staphylococcus chromogenes*, *Bacillus aquimaris*, *Staphylococcus arlettae*, and *Staphylococcus sciuri*, were identified. According to the phylogenetic tree constructed by the neighbor-joining algorithm, a close relationship was observed between the species of two genera, *Staphylococcus* and *Bacillus*, while the relationship was distant for the genus *Pantoea* (Figure 2(a)).

### 3.3. *Culex gelidus*

Overall, a total of nine bacterial species were isolated (*Staphylococcus sciuri*, *Staphylococcus warneri*, *Moraxella osloensis*, *Enterobacter* sp., *Klebsiella michiganensis*, *Staphylococcus hominis*, *Bacillus megaterium*, *Staphylococcus saprophyticus*, and *Streptomyces* sp.). These were different from the isolates observed form other two mosquito species in this study. It inferred relatively a distant phylogenetic relationship to *K. michiganensis*, *Enterobacter* sp., and *M. osloensis* (Figure 2(b)).

### 3.4. *Mansonia annulifera*

The lowest bacterial diversity was observed from *Ma. annulifera* consisting of only *Bacillus niacin*, *Cedecea neteri*, *Micrococcus luteus*, *Staphylococcus chromogenes*, *Lysinibacillus sphaericus*, and *Bacillus licheniformis*. However, of the six bacteria species identified, five of them were detected only from *Ma. annulifera*, while *S. chromogenes* was observed from *Cx. tritaeniorhynchus* (Table 1). It seems that there is a considerable difference between the gut bacteria in *Ma. annulifera* compared to the other two mosquito species investigated. *Bacillus niacini* and *B. licheniformis* shared a relatively close relationship, while *C. neteri* was having a distant phylogenetic relationship with the all other recorded species (Figure 2(c)).

### 3.5. Joint Phylogenetic Affiliation of the Midgut Bacterial Isolates in Three Selected Species

**Figure 3:**
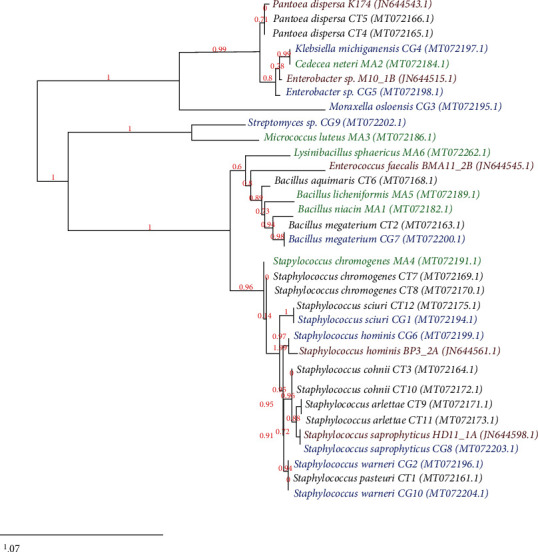
Phylogram showing the phylogenetic affiliation of bacterial isolates in the midgut of the three selected mosquito species (*Culex tritaeniorhynchus*, *Culex gelidus*, and *Mansonia annulifera*) along with reference sequences of some mid gut bacterial isolated from *Cx. quinquefasciatus* mosquito collected from India (Chandel et al., 2013).

The ML trees estimated from gut microbial sequence alignments showed *Moraxella osloensis* on a long branch and seemed to cluster separately from the majority of the remaining sequences belonging to phylum *Proteobacteria* (Figure 3). Phylum *Firmicutes*, on the other hand, was very compact and contained very short branches. Family *Staphylococcaceae* showed the most variation, with several distinct clusters of sequences. The genetic distance between same gut microbial species (*Pantoea dispersa*, *Staphylococcus chromogenes*, and *Staphylococcus hominis*) differed between host mosquito species in which they were isolated (*Cx. tritaeniorhynchus* and *Ma. annulifera*) (e.g., 0.347 for *Pantoea dispersa*) (Figure 3).

Genetic distance between some gut microbial strains varied even within the same host mosquito species in which they were isolated (e.g., *Staphylococcus arlettae* isolated from *Cx. tritaeniorhynchus*) (Figure 3). No significant difference was observed for the genetic distances of the same gut microbial strain isolated from the same mosquito species (within-group) or different mosquito species (between groups) (*P* < 0.001), and the genetic distance was not affected by the collection site of the host mosquito. The difference between Sri Lankan isolates from the reference sequences was also not significantly different with cutoffs at *P* ≤ 0.05.

### 3.6. Diversity of Mosquito Microbiota

The Shannon diversity indices revealed that the gut microbiota of *Cx. gelidus* was the highest diverse followed by *Cx. tritaeniorhynchus*. Simpson's diversity, Margalef's diversity, and Menhinick's diversity indices also advocated the above observation. The diversity indices scored by each mosquito species are given in Table 2.

### 3.7. Variation in Midgut Bacterial Communities across Mosquito Species

The midgut bacteria analysis among the three mosquito species studies were found to be quite similar. The most abundant midgut microbial species were associated with phylum *Firmicutes* with 87.5%, 55.6%, and 66.7% in *Cx. tritaeniorhynchus*, *Cx. gelidus*, and *Ma. annulifera*, respectively.

Other observed phyla found in all field-derived strains of mosquitoes were *Proteobacteria* (12.5%, 33.3%, and 16.7%, respectively, for the three above mosquito species) and *Actinobacteria* (0%, 11.1%, and 16.7%, respectively).

**Figure 4:**
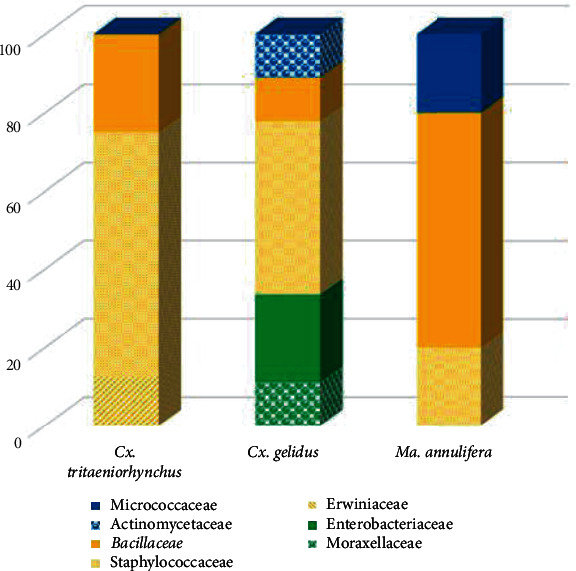
Relative abundances of bacterial families (7) of gut microbes present within three studied mosquito species collected at different sites by the 16S rRNA PCR product sequence analysis.

The relative abundance of families of gut microbial species present within three different mosquito species revealed that *Staphylococcaceae* is the most abundant gut microbial family, with 62.5%, 44.4%, and 20.0%, respectively, for three mosquito species followed by family *Bacillaceae* (25.0%, 11.1%, and 60.0%, respectively) and *Enterobacteriaceae* (0%, 22.2%, and 0%, respectively) (Figure 4).

Chi-square analysis revealed that the relative distribution of midgut microbes in different mosquito species differed significantly among three selected mosquito species (*χ*^2^ = 486.091; *df* = 36; *P* ≤ 0.001). Midgut microbiota in *Cx. tritaeniorhynchus* and *Cx. gelidus* indicated a similarity of 21.51%, while *Ma. annulifera* shared a similarity of 6.74% with the cluster of above two species.

**Figure 5:**
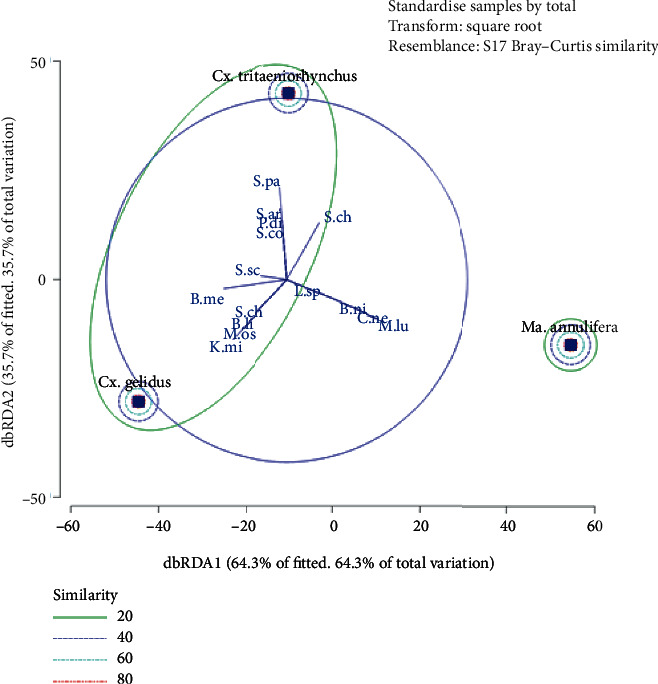
dbRDA plot for distribution of midgut microbiota in *Culex tritaeniorhynchus*, *Culex gelidus*, and *Mansonia annulifera.*

The midgut microbe assemblage among three mosquito species formed subclusters based on the Bray Curtis similarity. The midgut microbiota in *Cx. tritaeniorhynchus* and *Cx. gelidus* indicated similarity of 21.51%, while *Ma. annulifera* shared a similarity of 6.74% with the cluster of above two species. The dbRDA 1 and dbRDA 2 explained that 64.3% and 35.7% of the total variation of gut microbes in three mosquito species (Figure 5).

## 4. Discussion

Microbial symbionts in the insects exist at different organs such as the gut, ovaries, Malpighian tubules, and hemocoel [48]. The microbiota in the gut is of precise interest because it is the first contact point between the parasites and epithelial surfaces [49]. The prevalence of insect gut microflora especially in mosquitoes has been investigated through classical culture-based methods or metagenomic based on 16S rRNA gene sequencing [50–52]. The bacterial composition of mosquitoes sampled from natural habitats is highly variable but often contain a core microbiome that is dominated by a small number of taxa. However, it may vary subject to the insect species, geographical origin, ecological niche, source of food, and gender [50, 51, 53].

This microbiota can influence the capacity of insects to transmit disease-causing pathogens through various mechanisms. Therefore, understanding the interaction between vectors with their microbiota and transmitting pathogens may be useful to limit disease transmissions by exploring the potential role of microbiota in modulating infections that could lead to alternative disease control strategies [53]. Paratransgenesis is one such approach that controls the transmission of pathogens by arthropod vectors using gut microflora. This approach consists of the use of genetically altered symbiotic bacteria that secrete effector molecules that kill the infectious agent, disturb the reproduction, make the vector less competent, or reduce the life span of the host. However, this needs screening of microbiota among locally available disease-transmitting vectors using culture-dependent methods since the availability of gut microflora may vary with the geographical location [19].

The studies have carried out to screen the midgut microflora in mosquito species under different genera including *Aedes*, *Anopheles*, *Culex*, and *Mansonia*. However, only a few studies have been conducted to evaluate the prevalence of gut microflora in *Cx. tritaeniorhynchus*, *Cx. gelidus*, and *Ma. annulifera.* A study conducted by Abalain-Colloc et al. [54] has reported the presence of one microbiota species, *Spiroplasma taiwanense*, from the midgut of field-collected *Cx. tritaeniorhynchus* in Taiwan. This is the only available information regarding the midgut microbes of this species, whereas available information is limited for *Cx. gelidus* and *Ma. annulifera*. Even after 31 years, only a few studies have contributed to compensate for the above limitation, which may be grossly inadequate to get an idea about the diversity of gut microflora of the importance of pursuing control strategies. Thus, the present study contributes to filling the knowledge gap regarding the prevalence of gut bacterial flora in the above mosquito species.

Examination of 16S ribosomal RNA amplicons from culturable microflora isolated from the midgut of adult female *Cx. quinquefasciatus* from India revealed the presence of 83 bacterial species under 31 bacterial genera [19]. All species identified were fit into three Phyla: *Proteobacteria* (37 species), *Firmicutes* (33 species), and *Actinobacteria* (13 species). Similarly, the present study has also isolated the midgut bacterial species that belonged to the same three phyla mentioned above. These three bacterial phyla have commonly been recorded in the gut of many insect species including mosquitoes [12, 14, 16].

However, the diversity of microbes in Phylum *Actinobacteria* was comparatively lower (only 2 species) compared to the other two Phyla (*Proteobacteria* and *Firmicutes*). About 66.4% and 23.14% of the total isolated bacteria in *Cx. tritaeniorhynchus* belonged to genera *Staphylococcus* and *Bacillus*, respectively, which represented around 89.54% of the total bacterial population. None of the species was detected under phylum *Actinobacteria* from *Cx. tritaeniorhynchus*. Some of the *Staphylococcus* species isolated from *Cx. tritaeniorhynchus* and *Cx. gelidus* during the present study, namely, *S. cohnii*, *S. pasteuri*, *S. saprophyticus*, *S. arlettae*, *S. hominis*, and *S. warneri*, had also been recorded previously among the wild-caught *Aedes albopictus* in India [55]. Therefore, these species may coexist among mosquito species at different geographical isolates, and there could be alterations among the gut microflora in mosquitoes.

The presence of different microbial species as midgut bacteria in three mosquito species could be recognized as the reason for the separation of two distinct clusters in the dbRDA analysis. However, *S. chromogenes* was found as a common bacterial species between *Cx. tritaeniorhynchus* and *Ma. annulifera.* In addition, *S. sciuri* and *B. megaterium* were common in both *Cx. tritaeniorhynchus* and *Cx. gelidus*. The occurrence of *C. neteri*, *B. niacin*, *L. sphaericus*, and *B. licheniformis* was limited only to *Ma. annulifera*. This could be identified as another possible reason for the separation of midgut bacteria in *Ma. annulifera* as a single cluster.

There are some evidence on the usage of gut microbacteria available in the insects for control approaches in the means of vector suppression, inhibition of parasite/pathogenic development in the invertebrate hosts, oviposition attractants in the development of lethal ovitraps, and transformation carriers in expressing molecules. Apart from mosquitoes, achievements were made from kissing bug gut inhabiting microbe, *Rhodococcus rhodnii*, for the prevention of the Chagas 4disease and sand fly gut inhabiting microbe, *Enterobacter cloacae*, for reducing *Leishmania* parasite transmission [56]. *Bacillus megaterium* and *B. licheniformis* recorded during the present study have been identified previously as candidates for paratransgenesis in *Phlebotomus papatasi* [57] while *S. saprophyticus* detected from *Cx. gelidus* is used as a strong oviposition inducer for gravid *P. papatasi* [58]. In addition, some *Enterobacter* and *Klebsiella* species have been previously documented on the ability to reduce the intensity and prevalence of *Plasmodium falciparum* infection [59]. Furthermore, *Klebsiella* sp. serves as an attractant for the selection of oviposition habitat, and it nourishes the most fragile larval stage, first instars to more resilient second instars [60].

Besides, *Enterobacter* sp., *Klebsiella michiganensis*, and *Pantoea dispersa* that were recorded during the present investigation have also been identified as potential candidates for paratransgenesis in *Aedes* mosquitoes [55]. *Lysinibacillus sphaericus* isolated from *Ma. annulifera* has previously been isolated from *An. maculipennis* in Iran and revealed as a potential paratransgenic bacteria to control malaria transmission [61]. Therefore, exploring the feasibility of using these identified bacteria species for the control of different disease vectors in terms of their ability to inhibit the disease pathogen, as paratransgenesis candidates and oviposition attractant/inducer, is of paramount importance for a country like Sri Lanka.

The composition of gut microflora varies with the host defense and immune mechanisms in disease vectors. Recent investigations suggest the significance of microbiota in insects by sustaining its basal immune activity and immune priming [62–64]. Further, the microbiota can modulate the mosquito immune response and influence vector competence to human pathogens. For instance, elimination of the major midgut bacteria in *An. gambiae* and *Ae. aegypti* through antibiotics may increase the susceptibility to *P. falciparum* and dengue virus (DENV) infections, respectively [6, 65]. In addition, bacteria can also influence the mosquito competence by impairing the pathogenic infection through competition for resources or secretion of antipathogen molecules [66–68]. It has been noticed that during the chikungunya infection in *Aedes* mosquitoes, the abundance of bacteria under *Enterobacteriaceae* family is elevated [13]. Therefore, it is deduced that the changes in gut microflora may represent the occurrence of disease pathogens which may be used as reflective organisms to identify the presence of disease pathogens in the wild population of vectors. Further, this phenomenon can be used to suppress vector population through modulation of gut microflora by introducing certain field-derived bacteria into the mosquito gut through an artificial feeding which may increase mosquito resistance to the pathogen [53].

Modification of bacteria species in recognition of pathogens through pattern recognition receptors (PRRs), immune pathway activation results in the systemic production of anti-microbial peptides (AMPs), and other antipathogen immune effectors, as well as stimulating immune defense mechanisms, is one of the applications [53]. *Asaia* sp. which is isolated from the midgut of *Ae. aegypti* was found to be expressing antipathogenic molecules within the mosquito [56]. The genetically modified *Asaia* can be successfully transformed into adults through sugar or blood meals and thereby can be vertically transmitted horizontally or cofeeding [69]. The Anti-Plasmodium effect or molecules can be displayed by genetically modified *Escherichia coli* in the gut of *An. stephensi* mosquitoes by producing effector molecules for inhibition of *P. berghei* development inside the mosquito [70].

*Pantoea dispersa* (phylum *Proteobacteria*) was identified from the midgut of adult *Cx. tritaeniorhynchus* during the present study with a 10.44% of relative abundance. A study has indicated that another symbiotic bacterium in the genus *Pantoea* (*Pantoea agglomerans*) could express and secrete the anti-Plasmodium effect or proteins (SM1, anti-Pbs21, and PLA2) [71]. Further, genus *Pantoea* is a possible candidate for paratransgenesis and is known for the vector competence of *Ae. albopictus* as well [51]. Moreover, genus *Pantoea*, which was recorded from *Cx. tritaeniorhynchus*, exhibits the transstadial and horizontal transmission properties [72]. Therefore, in paratransgenesis application, the modified bacteria could be provided with the larval diet which can also be transmitted to the adult stage through transstadial and horizontal mechanisms. Therefore, the species in the genus *Pantoea* may be a suitable candidate for the paratransgenesis approach. However, the feasibility of the identified species from the present study for such intervention needs more investigations.

The present study suffers from some limitations. The composition of gut microbiota in different mosquito species varies as a result of complex interactions between environmental characteristics and developmental stages, physiological status, and feeding behaviour [73] which may influence the findings in the present study. Therefore, cattle-baited net traps were set up for mosquito collections with similar environmental settings such as closer to marshy lands, human habitation, and live-stock sheds to minimize the potential bias in the diversity of midgut bacteria that may be caused due to environmental variables. Host-related factors such as nutrition also influence microbiota composition [17]. For instance, blood meal induces an overall decrease of microbiota richness during the first 24 hours after the blood meal [74, 75]. However, the bacterial population increases substantially 48 h after a blood meal. Changes in the composition of the gut microbiota following a blood meal may be due to the oxidative stress associated with the catabolism of the blood meal [12, 76]. Further, some studies have emphasized that sugar feeding also influences the diversity of midgut microbiota in mosquitoes [77]. Therefore, midguts of non-blood-fed field-caught adult female mosquitoes were taken for dissections under sterile conditions for the isolation of the midgut bacterial population. Surface sterilized mosquitoes were taken for dissections immediately within 6-8 hours after field collections. Further, during the rearing of mosquitoes at the insectary, no artificial feeding of blood or sugar source was provided.

When using a culture-dependent approach, only 20% of environmental bacteria can be grown on a growth medium [78]. Thus, the composition of the microbiota is not a direct reflection of richness and abundance of the whole gut bacterial community which rises as a limitation of the culture-dependent microflora analysis. The development of future perspectives for paratransgenesis approach in Sri Lanka is interesting and necessary to examine the potential success of this method. To that end, *P. dispersa* can be proposed as the best candidate, and strains of nonpathogenic species in *Bacillaceae* family such as *B. megaterium*, *B. niacini*, *B. licheniformis*, and *Lysinibacillus sphaericus* were identified as other potential candidates for paratransgenesis. Therefore, while filling the lack of updated information on symbiotic bacteria in the midgut of *Cx. tritaeniorhynchus*, *Cx. gelidus*, and *Ma. annulifera* mosquitoes, the present data strongly encourages further investigations to explore the potential usage of these microbes through paratransgenic approach in consideration with ecological and other safety concerns.

## 5. Conclusion

This is the first attempt to explore the midgut microbiota of *Culex tritaeniorhynchus*, *Culex gelidus*, and *Mansonia annulifera* in Sri Lanka. Since there is only a few published information on the midgut microflora of *Culex* mosquitoes and the absence of any study for *Mansonia annulifera* mosquitoes, the present study provides fundamental information to the literature. The relative distribution of midgut microbes in different mosquito species differed significantly among the three studied mosquito species. The gut microbiota of *Cx. gelidus* was the highest diverse followed by *Cx. tritaeniorhynchus* and *Ma. annulifera*.

For paratransgenic applications, the feasibility of *P. dispersa* could be evaluated, and the suitability of nonpathogenic species in *Bacillaceae* family such as *B. megaterium*, *B. niacini*, *B. licheniformis*, and *Lysinibacillus sphaericus* should be further investigated. Therefore, the present data strongly encourage further investigations to explore the potential usage of these microbes through the paratransgenic approach which is a novel eco-friendly vector control strategy.

## Figures and Tables

**Table 1:** The occurrence of different bacterial species in *Culex tritaeniorhynchus*, *Culex gelidus*, and *Mansonia annulifera.*

Phylum	Family	Bacterial species (Accession No.)	Percentage occurrence (95% CI^b^)		
			*Cx. tritaeniorhynchus*	*Cx. gelidus*	*Ma. annulifera*
*Proteobacteria*	*Moraxellaceae*	*Moraxella osloensis* (MT072195)	ND	5.56 (0.025-0.119)	ND
	*Enterobacteriaceae*	*Klebsiella michiganensis* (MT072197)	ND	18.06 (0.117-0.267)	ND
		*Enterobacter* sp. (MT072198)	ND	11.11 (0.063-0.187)	ND
		*Cedecea neteri* (MT072184)	ND	ND	21.05 (0.142-0.3)
	*Erwiniaceae*	*Pantoea dispersa* (MT072165/MT072166)	10.44 (0.058-0.179)	ND	ND

*Firmicutes*	*Staphylococcaceae*	*Staphylococcus sciuri* (MT072175/MT072194)	6.71 (0.032-0.133)	8.33 (0.043-0.154)	ND
		*Staphylococcus warneri* (MT072204/MT072196)	ND	5.56 (0.025-0.119)	ND
		*Staphylococcus hominis* (MT072199)	ND	10.42 (0.058-0.179)	ND
		*Staphylococcus saprophyticus* (MT072203)	ND	9.72 (0.053-0.171)	ND
		*Staphylococcus chromogenes* (MT072169/MT072170/MT072191)	16.42 (0.104-0.248)	ND	11.84 (0.069-0.196)
		*Staphylococcus pasteuri* (MT072161)	20.89 (0.141-0.298)	ND	ND
		*Staphylococcus cohnii* (MT072164/MT072172)	8.95 (0.047-0.161)	ND	ND
		*Staphylococcus arlettae* (MT072171/MT072173)	13.43 (0.081-0.214)	ND	ND
	*Bacillaceae*	*Bacillus megaterium* (MT072163/MT072200)	12.69 (0.075-0.206)	20.83 (0.140-0.298)	ND
		*Bacillus niacini* (MT072182)	ND	ND	15.78 (0.099-0.242)
		*Bacillus aquimaris* (MT072168)	10.45 (0.058-0.179)	ND	ND
		*Bacillus licheniformis* (MT072189)	ND	ND	22.36 (0.153-0.315)
		*Lysinibacillus sphaericus* (MT072262)	ND	ND	1.32 (0.003-0.059)

*Actinobacteria*	*Actinomycetaceae*	*Streptomyces* sp. (MT072202)	ND	10.42 (0.058-0.179)	ND
	*Micrococcaceae*	*Micrococcus luteus* (MT072186)	ND	ND	28.00 (0.198-0.371)

^a^All bacterial species were identified based on a % identity higher than 99% CI. ^b^Confidence interval; ND: not detected.

**Table 2:** Diversity indices and total taxa of midgut bacterial isolates of *Culex tritaeniorhynchus*, *Culex gelidus*, and *Mansonia annulifera.*

Criteria	Mosquito species		
	*Cx. tritaeniorhynchus*	*Cx. gelidus*	*Ma. annulifera*
Total taxa identified	8	9	6
Dominance	0.38	0.41	0.21
Simpson's diversity index	0.86	0.87	0.80
Shannon's diversity index	17.08	20.60	8.25
Evenness	5.80	7.00	2.80
Margalef's diversity index	3.29	3.71	2.13
Menhinick's diversity index	0.69	0.75	0.58

## Data Availability

The datasets supporting the conclusions of this article are included in the manuscript.
